# Structural analysis of peptide identified from the 2KRR domain of the nucleolin protein with a c-Myc G4 structure using biophysical and biochemical methods

**DOI:** 10.1039/d4ra02785j

**Published:** 2024-07-19

**Authors:** Sarvpreet Kaur, Nikita Kundu, Taniya Sharma, J. Shankaraswamy, Sweta Singh, Sarika Saxena

**Affiliations:** a Amity Institute of Biotechnology, Amity University Uttar Pradesh Noida 201313 India ssaxena1@amity.edu sarikaigib@yahoo.co.in +91 120-4735600; b Department of Fruit Science, College of Horticulture, Mojerla, Sri Konda Laxman Telangana State Horticultural University 509382 Telangana India; c Institute of Nuclear Medicine and Allied Sciences (INMAS), DRDO Brig. S. K. Mazumdar Marg Delhi-110054 India

## Abstract

For the first time, the c-Myc G4 structure is reported to be stabilized by binding of the peptide (derived from the 2KRR domain of the nucleolin protein called the Nu peptide) in the loop region of the G-quadruplex structure by stacking interactions. CD results showed the formation of parallel G4 structure in the presence of 100 mM Na^+^ or 100 mM K^+^ with the appearance of two isodichroic points at 229 nm, 254 nm and 252 nm in the presence of 100 mM Na^+^ or 100 mM K^+^, respectively. In addition, in UV thermal and CD melting studies, we observed drastic changes with an increase in the hyperchromicity at a DNA : peptide ratio of 1 : 50. On titrating the Nu peptide with c-Myc G4, we calculated the value of binding constant (*K*_a_) by plotting fluorescence intensity and DNA concentration as 0.1369 ± 0.008 μM and 0.1277 ± 0.073 μM in Na^+^ and K^+^, respectively, which confirms the strong association of Nu peptide with c-Myc G4. The Nu peptide showed preferential cytotoxicity against MDA-MB-231 cells with IC_50_ values of 5.020 μM and 5.501 μM after 72 and 96 hours. This approach suggests a novel strategy to target G4 structure using natural key peptide segments derived from G4 stabilizing protein.

## Abstract 2

The stabilization of c-Myc G4 structure is reported by binding of the peptide (derived from the 2KRR domain of the nucleolin protein called the Nu peptide) in the loop region of the G-quadruplex structure by stacking interactions.

## Graphical abstract

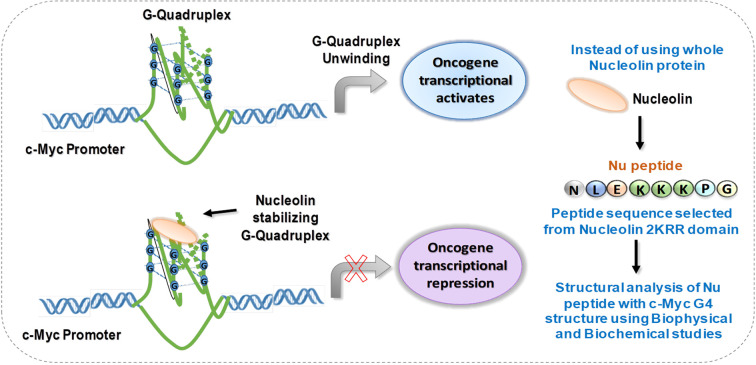

## 1 Introduction

The G-quadruplex (G4) structures are non-canonical secondary structures formed in guanine-rich regions of nucleic acids, particularly DNA. These structures are characterized by the stacking of planar arrays of four guanine bases (G-tetrads) held together by Hoogsteen hydrogen bonding. Initially discovered in telomeric DNA, G4 structures have since been identified in various regions of the human genome, including promoters, untranslated regions (UTRs), and intronic regions.^1,2^ The evolutionary stability of G4 structures suggests their functional importance. Genome-wide studies have revealed the prevalence of G4 motifs, particularly in the promoter regions of genes involved in cancer-related pathways, such as cell cycle regulation, proliferation, and apoptosis.^3^ Among these, the c-Myc oncogene has been extensively studied. The c-Myc promoter region contains a highly conserved G4-forming sequence that regulates c-Myc transcription.^4^ The structural stability of G4 structures makes them attractive targets for therapeutic intervention. Small molecules that stabilize G4 structures can inhibit the activity of telomerase and other G4-associated proteins, leading to potential anti-cancer effects.^5^ Additionally, peptide ligands have been developed to target G4 structures, offering a ligand-based approach to drug development.^4^

In this study, we focus on structurally studying a peptide identified from the 2KRR domain of the nucleolin protein with the c-Myc G4 structure. Understanding the interactions between this peptide and the c-Myc G4 could provide insights into developing of novel therapeutics *via* targeting c-Myc regulation. The search for G-quadruplex (G4)-binding ligands has become a significant focus in recent years. In this study, we identified a short peptide from the nucleotide-binding domains of the G-quadruplex-binding nucleolin protein for the first time. Additionally, we aimed to create a peptide library of known G-quadruplex-binding proteins to help identify small peptides. Our approach involved collecting crystal structures or NMR structures of G4s from the NCBI database, including those from human telomeres and various human cancer proto-oncogenes, as well as the structure of G4 binding proteins, such as the NCL-2KRR domain. Next, we docked the selected protein domains with selected G4s and created a peptide library to identify short peptides that could perform transcriptional silencing of proto-oncogenes and regulate the structure of telomeric G4s to alter the binding of telomerase enzyme (unpublished data and patent filed; patent ID: 202 211 008 538). Recently, many proteins with binding affinity to G-quadruplexes have been identified. One of the early known G-rich regions, the human telomeric sequence (TTAGGG)_*n*_, is recognized by many proteins that can modulate telomerase activity. Sequences with the potential to form G-quadruplexes are often located in the promoter regions of various oncogenes. The NHE III1 region of the c-Myc promoter has been shown to interact with nucleolin protein, as well as other G-quadruplex-binding proteins.^6^ Nucleolin is a multifunctional protein known to interact with various RNA and DNA structures, including hairpin RNA, double-stranded DNA, and G-quadruplex structures. In the context of the c-Myc gene, nucleolin has been demonstrated to bind to the single-stranded and G-quadruplex conformations in the G-rich NHE III1 region of the promoter. Studies using a filter binding assay have demonstrated that nucleolin exhibits a higher affinity for the G-quadruplex structure compared to other conformations in this region, such as the C-rich strand or the double-stranded DNA. This specific interaction suggests a potential regulatory role of nucleolin in the c-Myc gene expression, particularly through its binding to the G-quadruplex structure in the NHE III1 region.^6^ Nucleolin is a nucleolar phosphoprotein that is highly expressed in proliferating cells with multiple roles in ribosome biogenesis,^7^ chromatin remodelling,^8^ transcription,^9^ G-quadruplex binding,^10^ and apoptosis.^11^ Interestingly, nucleolin-hnRNP D heterodimer has been reported to bind to G-quadruplex structures.^12^ It was shown that overexpression of nucleolin can significantly inhibit the c-Myc promoter-driven transcription as measured by luciferase activity in MCF10A cells.^13^ Nucleolin binds *in vitro* to the c-Myc G-quadruplex structure with high affinity and selectivity as compared with other known quadruplex structures. In addition, nucleolin facilitates the formation of the c-Myc G-quadruplex structure and increases its stability. Importantly, it was also revealed that nucleolin binds to the c-Myc promoter *in vivo*.^14^

In the present study, we investigated the interaction between a selected peptide identified from the 2KRR domain of the nucleolin protein and human c-Myc G4 under physiological ionic conditions (100 mM Na^+^ or 100 mM K^+^). The peptide's interaction with c-Myc G4 was determined using biophysical and biochemical methods. Due to the complexities and limitations of using the entire G4-protein for interaction studies—such as its complex structure and the difficulty in targeting G4—it is preferable to focus on small molecules to target G4s. Therefore, we identified the specific domain of nucleolin that binds to the G4 and used that 8-mer sequence for interaction studies. Using a peptide, instead of a synthetic molecule, as a ligand is advantageous because peptides are promising next-generation drug molecules due to their high specificity and potency, biocompatibility, and ability to perform diverse biological functions. They are easier to synthesize and modify, can target complex structures and benefit from improved delivery methods. Peptides generally have lower toxicity and receive increasing regulatory approvals, making them a versatile and powerful option for developing new therapeutics. Circular dichroism (CD) data suggested that c-Myc G4 folds into parallel G-quadruplexes, and two isodichroic points appeared at 229 nm and 254 nm in the presence of 100 mM Na^+^ and at 252 nm with 100 mM K^+^. In addition, we observed drastic changes in the DNA-to-peptide ratio of 1 : 50 with an increase in hyperchromicity in UV thermal and CD melting studies. On titrating the Nu peptide with an increasing concentration of pre-formed c-Myc G4, we plotted the fluorescence intensity *versus* DNA concentration and calculated the value of binding constant (*K*_a_) to be 0.1369 ± 0.008 μM and 0.1277 ± 0.073 μM in Na^+^ and K^+^, respectively, which confirms the strong association of Nu peptide with c-Myc G4. The Nu peptide shows preferential cytotoxicity with IC_50_ values of 5.020 μM and 5.501 μM after 72 and 96 hours in MDA-MB-231 cells. This approach suggests a novel strategy to target the G4 structure using natural key peptide segments derived from the G4 stabilizing protein. This study will shed light on understanding the role of G-quadruplex structures and interactions of peptide complexes in cellular processes and will highlight the possibility of peptides being used as next-generation drug molecules for cancer patients.

## 2 Materials and methods

### 2.1 DNA and peptide

A PAGE-purified DNA oligonucleotide [c-Myc promoter sequence-5′-TGAGGGTGGGTAGGGTGGGTAA-3'] and an HPLC-purified peptide [NLEKKKPG] were procured from Helix Biosciences. Single-strand DNA oligonucleotide concentrations were determined by measuring absorbance at 260 nm at high temperature using a Shimadzu 1800 Spectrophotometer (Shimadzu, Tokyo, Japan) connected to a thermoprogrammer. Extinction coefficients for single strands were calculated based on mononucleotide and dinucleotide data using the nearest neighbor approximation.

### 2.2 Circular dichroism spectroscopy

CD spectra were conducted using a JASCO-715 spectro-polarimeter with a quartz cuvette of 1 cm path length. Spectra were recorded within the wavelength range of 200–350 nm at a scanning rate of 100 nm min^−1^. Before measurement, samples were heated to 95 °C in a water bath, slowly cooled to room temperature, and further incubated overnight at 4 °C to prevent non-equilibrium structures. The average scans of DNA samples were subtracted from the buffer scan, and the data were normalized based on the concentration of DNA strands and path length of the cuvette.

### 2.3 Thermal melting analysis

UV absorbance measurements of various samples were conducted using a Shimadzu 1800 spectrophotometer equipped with a temperature controller. Melting curves of DNA structures were obtained by measuring UV absorbance at either 260 or 295 nm in 30 mM sodium cacodylate buffer (pH 7.4) containing 0.5 mM EDTA with either 100 mM NaCl or 100 mM KCl in the presence or absence of Nu peptide at DNA : peptide ratios of 1 : 0, 1 : 2, 1 : 5, 1 : 20, and 1 : 50. The melting temperatures (*T*_m_) for 4 μM DNA structures were determined from the UV melting curves using a heating rate of 0.5 °C min^−1^ using 8-cell stoppered quartz cuvette of 1 cm path length. Before measurement, samples were heated to 95 °C in a water bath, slowly cooled to room temperature, and later incubated overnight at 4 °C to prevent non-equilibrium structures.^15,16^

### 2.4 Native gel electrophoresis

In the native gel experiment, a 15% (w/v) polyacrylamide gel was employed. Samples were prepared in 30 mM sodium cacodylate buffer (pH 7.4) containing 0.5 mM EDTA, along with either 100 mM NaCl or 100 mM KCl. The samples were heated to 95 °C in a water bath, slowly cooled to room temperature, and later incubated overnight at 4 °C. The running buffer, tris-borate-EDTA (TBE), at pH 7.4 also contained the same concentration of salt and EDTA as the oligonucleotide sample in the gel. The experiment was conducted in a cold room at a constant voltage of 50 V. A tracking dye comprising a 1 : 1 mixture of glycerol and orange-G was used to monitor the movement of DNA oligonucleotides in the gel. Finally, the gel was stained using silver staining and imaged using a Gel-Doc system (Biorad, Gurgaon, Haryana, India).

### 2.5 Fluorescence measurements

Fluorescence experiments were conducted using a JASCO FP 8300 spectrofluorometer (JASCO, Tokyo, Japan) at 25 °C with a 1 cm path-length quartz cuvette. A peptide concentration of 4 μM was prepared in 30 mM sodium cacodylate buffer (pH 7.4) containing either 100 mM NaCl or 100 mM KCl, along with 0.5 mM EDTA. The peptide solution was titrated with an equimolar concentration of pre-formed c-Myc G-quadruplex. The temperature of the cell holder was controlled using a JASCO ETC-273T temperature controller. Samples were prepared using the same procedure. Excitation and emission slit widths were set to 5 nm, with excitation at 275 nm and emission recorded in the range of 270 nm to 500 nm.

### 2.6 *In vitro* cytotoxic assay of Nu peptide

MDA-MB-231 (epithelial) cells were obtained from the National Centre for Cell Sciences (NCCS), Pune, India and cultured in Roswell Park Memorial Institute Medium (RPMI) from Sigma-Aldrich, USA. The cells were grown in RPMI medium supplemented with 10% Fetal Bovine Serum (FBS) and 1% HEPES, along with 1% penicillin/streptomycin, in a 5% CO_2_ humidified incubator at 37 °C. The Nu peptide was completely dissolved in the RPMI medium to obtain a concentration of 40 mM. The cells were maintained under standard cell culture conditions. After 24 hours, the growth medium was replaced, and freshly prepared Nu peptide stock was serially diluted eleven times by two-fold dilution (40 mM, 20 mM, 10 mM, 5 mM, 2.5 mM, 1.25 mM, 625 nM, 312 nM, 150 nM, 78 nM, 39 nM). Each concentration was added to the respective wells in triplicates in a total volume of 100 mL, followed by incubation under standard conditions. Non-treated control cells were also maintained under the same conditions to serve as a comparison for growth inhibition. After 72 and 96 hours of treatment, the contents of all wells were removed, and 20 mL of reconstituted MTT (Sigma) was added. Following a 2 h dark incubation in a 5% CO_2_ humidified incubator, the supernatant was removed, and 100 mL of the MTT solubilization solution was added. The plates were then placed in a shaking incubator at 37 °C to solubilize formazan crystals. Absorbance was measured at 570 nm using a microplate reader, and the IC_50_ value was determined using GraphPad Prism.

## 3 Results and discussion

### 3.1 Binding model of c-Myc promoter G-quadruplex with Nu peptide

**Fig. 1:**
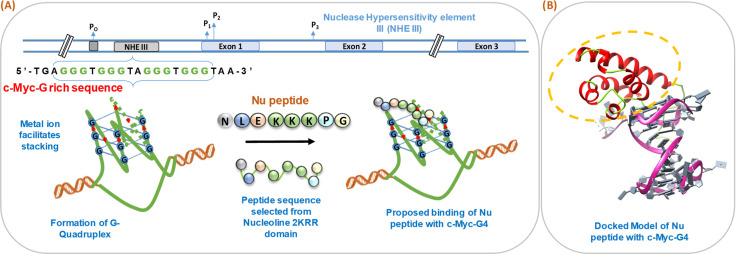
Schematic representation of the formation of human c-Myc-G-quadruplex from the nuclease hypersensitivity element III (NHE III) located upstream of the P1 promoter and proposed binding of Nu peptide to c-Myc G4 (A). Molecular docking model of c-Myc-G4 (6AU4) with Nu peptide where the binding of Nu peptide to the loop region of G4 is shown (B).

We have schematically shown the arrangement of G-quadruplex forming motifs located at the NHE1 site in the promoter region of the c-Myc gene (Fig. 1a). Next, we have shown the stages of the possible binding of Nu peptide in the loop region of c-Myc G4 structure in a stepwise manner. Fig. 1b shows the docking model of the Nu peptide with c-Myc G4 (6AU4), where it can be seen that the Nu peptide binds in the loop region. We proposed the mode of binding of the Nu peptide with the G4 structure with phosphate; the peptide contains three lysine residues, and thus, having a positively charged amino acid may allow it to bind to the G4 structures *via* phosphates. As the peptide sequence contains proline, it is suggested that the pyrrolidine ring of proline could interact with the bases in the loop region, potentially contributing to the stabilization of the G4 structure.

### 3.2 Structural analysis of human c-Myc promoter G-quadruplex with and without peptide under dilute conditions

**Fig. 2:**
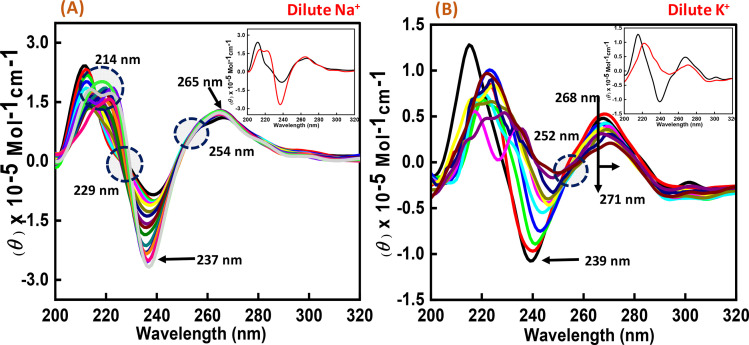
CD spectra of 6 μM human c-Myc-G4 in 30 mM sodium cacodylate buffer (pH 7.4) containing 0.5 mM EDTA, 100 mM NaCl (A) and 100 mM KCl (B) without any additive (black line) and titrated with an increasing concentration of Nu peptide (concentration of Nu used – 3 μg mL^−1^ to 1.12 mg mL^−1^). The inset graph displays the first and last titration curves, illustrating the structural changes observed at the end of the titration.

To perform the structural analysis of the G-quadruplex formed by the human c-Myc promoter sequence (5′-TGAGGGTGGGTAGGGTGGGTAA-3′), CD spectroscopy was performed in dilute cationic solutions of Na^+^ and K^+^ with and without the Nu peptide. The structure of each DNA strand was recorded in the presence of 30 mM sodium cacodylate buffer pH 7.4 and 100 mM Na^+^ and K^+^ in the presence and absence of Nu peptide (Fig. 2). CD spectra in 100 mM Na^+^ was characterized by a positive peak at 265 nm and a negative peak at 237 nm, which is typically observed for parallel G-quadruplex.^17–19^ The structure was then titrated with increasing concentration of Nu peptide to determine the conformational changes in G4 structure upon peptide binding. We observed a change in CD intensity at 237 nm, but a slight change in CD intensity at 265 nm was observed upon titration (concentration of Nu used – 3 μg mL^−1^–1.12 mg mL^−1^) with two isodichroic points at 229 nm and 254 nm. These changes in the intensity were not very significant; however, the presence of isodichroic points indicated that the peptide bound to the G4 structure but did not cause a significant change in the conformation of G4 in the presence of Na^+^. In contrast, in the presence of potassium ions (K^+^), the c-Myc promoter G4 structure displayed a characteristic circular dichroism (CD) spectrum, showing a strong positive peak at 268 nm and a negative peak at 239 nm. These peaks are indicative of a parallel G4 structure comprising a specific arrangement of nucleic acid bases. When the c-Myc G4 was titrated with increasing concentrations of Nu peptide, there were notable changes in the CD spectrum. The positive peak at 268 nm shifted to 271 nm, and there was a reduction in CD intensity at this wavelength. Additionally, the negative peak at 239 nm disappeared completely. These changes suggest a structural alteration in the c-Myc G4 upon interaction with the Nu peptide. An isodichroic point at 252 nm was observed during the titration. An isodichroic point is a wavelength at which the change in CD intensity due to the addition of the Nu peptide is balanced, indicating a transition between two distinct states of the G4 structure. This finding suggests that the Nu peptide binds strongly to the c-Myc G4 structure, where a stronger binding is induced by the potassium ions compared to sodium ions (Na^+^) by causing a conformational change in the G4 structure. Overall, these results indicate that the Nu peptide has the potential to interact with and stabilize the c-Myc G4 structure, particularly in the presence of potassium ions, which is further supported by UV thermal melting data.

### 3.3 CD melting studies of c-Myc promoter G4 with and without the nucleolin peptide

**Fig. 3:**
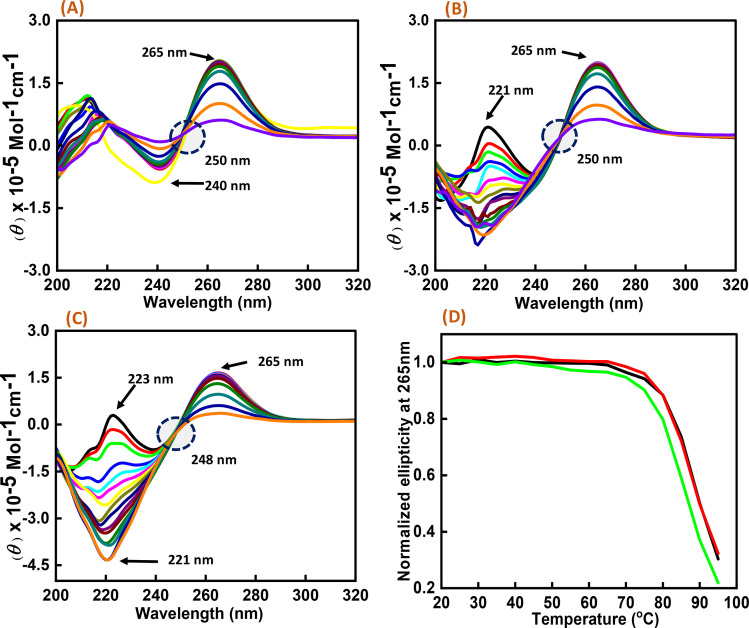
CD melting spectra of 6 μM human c-Myc-G4 in 30 mM sodium cacodylate buffer (pH 7.4) containing 0.5 mM EDTA, 100 mM KCl with different concentration of Nu peptide, (DNA : peptide) ratios of (1 : 0)-(A), (1 : 20)-(B) and (1 : 50)-(C) respectively and normalized CD melting curve at 265 nm of (DNA : p eptide) – (1 : 0)-(black), (1 : 20)-(red) and (1 : 50)-(green), respectively (D).

As significant changes were observed in c-Myc G4 with Nu peptide in the presence of K^+^, CD melting studies were performed on the c-Myc promoter G4 structure in the presence of K^+^ ion with and without the nucleolin peptide. Three different DNA : peptide ratios (1 : 0), (1 : 20), and (1 : 50) were prepared, and temperature-dependent CD spectra were recorded at temperature intervals of 5 °C, starting from 20 °C to 95 °C (Fig. 3). The change in the CD intensity against temperature was plotted. We did not observe any significant change in the *T*_m_ value, but the percentage of hyperchromicity increased upon increasing the peptide concentration. These CD melting results confirmed the binding of Nu peptide to c-Myc promoter G4 structure in the loop region and its stabilization was possibly due to the increase in stacking interaction.

### 3.4 Thermodynamic analysis of the human c-Myc promoter G4 structure with and without Nu peptide under dilute conditions

**Fig. 4:**
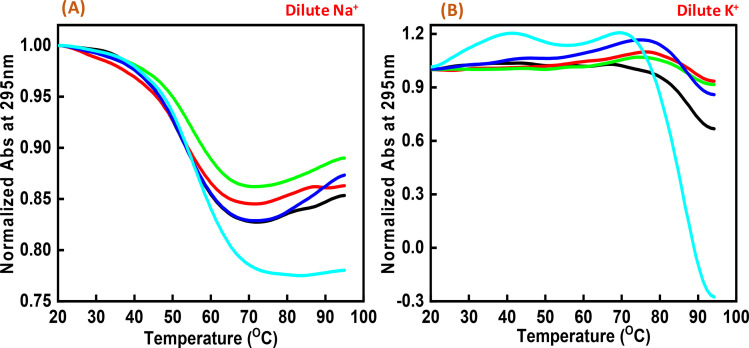
Normalized UV melting curves of 4 μM human c-Myc-G4 in 30 mM sodium cacodylate buffer (pH 7.4) containing 0.5 mM EDTA, 100 mM NaCl (A) and 100 mM KCl (B), c-Myc: Nu in DNA : peptide ratio of (1 : 0) (black), (1 : 2) (red), (1 : 5) (green), (1 : 20) (dark blue) and (1 : 50) (turquoise) at 295 nm.

Next, we explored the thermal stability of the c-Myc quadruplex structures with and without Nu peptide. Fig. 4 shows the normalized UV thermal melting profiles of 4 μM c-Myc promoter G4 in a buffer containing 100 mM NaCl and KCl in the presence and absence of Nu peptide at 295 nm. The different DNA : peptide ratios were used to record the changes in c-Myc G4 structure upon peptide binding at 1 : 0, 1 : 2, 1 : 5, 1 : 20 and 1 : 50, respectively. The melting temperature (*T*_m_) was evaluated by a curve-fitting procedure described previously.^15,16^Table 1 shows the *T*_m_ values for Na^+^ and K^+^ under dilute conditions.

**Table 1:** Melting temperature (*T*_m_) values for human c-Myc promoter G-quadruplex with and without the Nu peptide

DNA : peptide ratio	*T*_m_ (°C)	
	100 mM Na^+^	100 mM K^+^
1 : 0	51.8	87.6
1 : 2	52.5	87.6
1 : 5	54.0	87.6
1 : 20	54.8	87.6
1 : 50	55.5	88.5

Interestingly, these *T*_m_ values did not show much difference upon increasing the Nu peptide concentration, but drastic changes related to the increase in hyperchromicity were observed. This change was more significant in the case of K^+^ than for Na^+^. This indicated that the Nu peptide might bind with the loop region of c-Myc G4 structure in Na^+^ and K^+^ stabilizing the G4 structure upon an increase in the Nu peptide concentration, possibly by stacking interactions; these inferences are consistent with the CD melting results.

### 3.5 Native gel electrophoresis of the c-Myc promoter G4 with Nu peptide

**Fig. 5:**
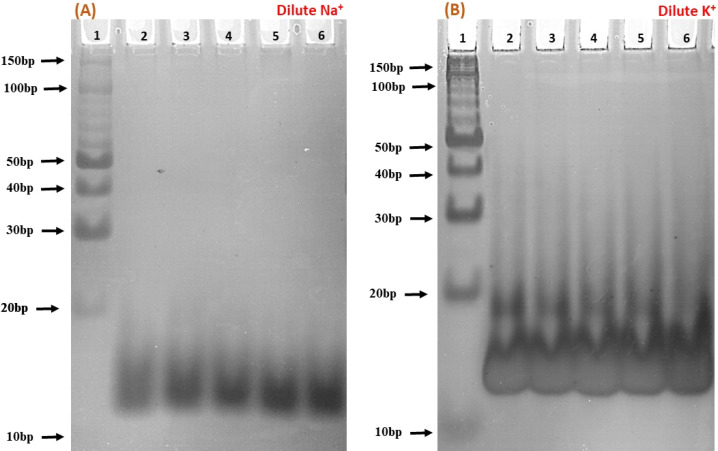
Native gel electrophoresis of 6 μM human c-Myc-G4 in 30 mM sodium cacodylate buffer (pH 7.4) containing 0.5 mM EDTA, 100 mM NaCl (A) and 100 mM KCl (B). Lane 1 has the 10 bp ladder; lane 2 has c-Myc : nucleolin-2KRR (1 : 50), lane 3 (1 : 20), lane 4 (1 : 5), lane 5 (1 : 2) and lane 6 (1 : 0), respectively.

To elucidate the molecular interactions between the c-Myc promoter G4 and Nu peptide, we conducted experiments using non-denaturing PAGE in the presence of 100 mM Na^+^ and K^+^ ions. This PAGE experiment allowed us to distinguish between the structures of the c-Myc promoter G4 alone and complex containing the c-Myc promoter G4-Nu peptide. The electrophoretogram presented in Fig. 5 depicts the structural changes in the c-Myc promoter G4 in the absence and presence of the Nu peptide. Lane 1 in the gel represents the 10 bp DNA ladder used as a reference for comparing electrophoretic mobility. A single prominent band between 10 to 20 bp was observed, indicating that the c-Myc promoter G4 adopts a unimolecular G-4 quadruplex structure. To further investigate the effect of Nu peptide on the migration of c-Myc promoter G4, different c-Myc : peptide ratios were prepared: (1 : 50) in lane 2, (1 : 20) in lane 3, (1 : 5) in lane 4, (1 : 2) in lane 5, and (1 : 0) in lane 6.

In the presence of Na^+^ (Fig. 5a), an increase in band intensity was observed from lane 2 to lane 6 with higher peptide concentrations. In Fig. 5b, in the presence of K^+^, a similar pattern was observed, but the band intensity was notably higher. Additionally, a minor band close to 20 bp began to appear with increasing peptide concentration, suggesting that peptide binding may have induced stabilization of the c-Myc promoter G4 structure, and the appearance of a new band may be due to the G4-peptide complex. This stabilization was more pronounced in K^+^ compared to Na^+^.

### 3.6 Exploration of Nu peptide binding to c-Myc promoter G-quadruplex by fluorescence spectroscopy

**Fig. 6:**
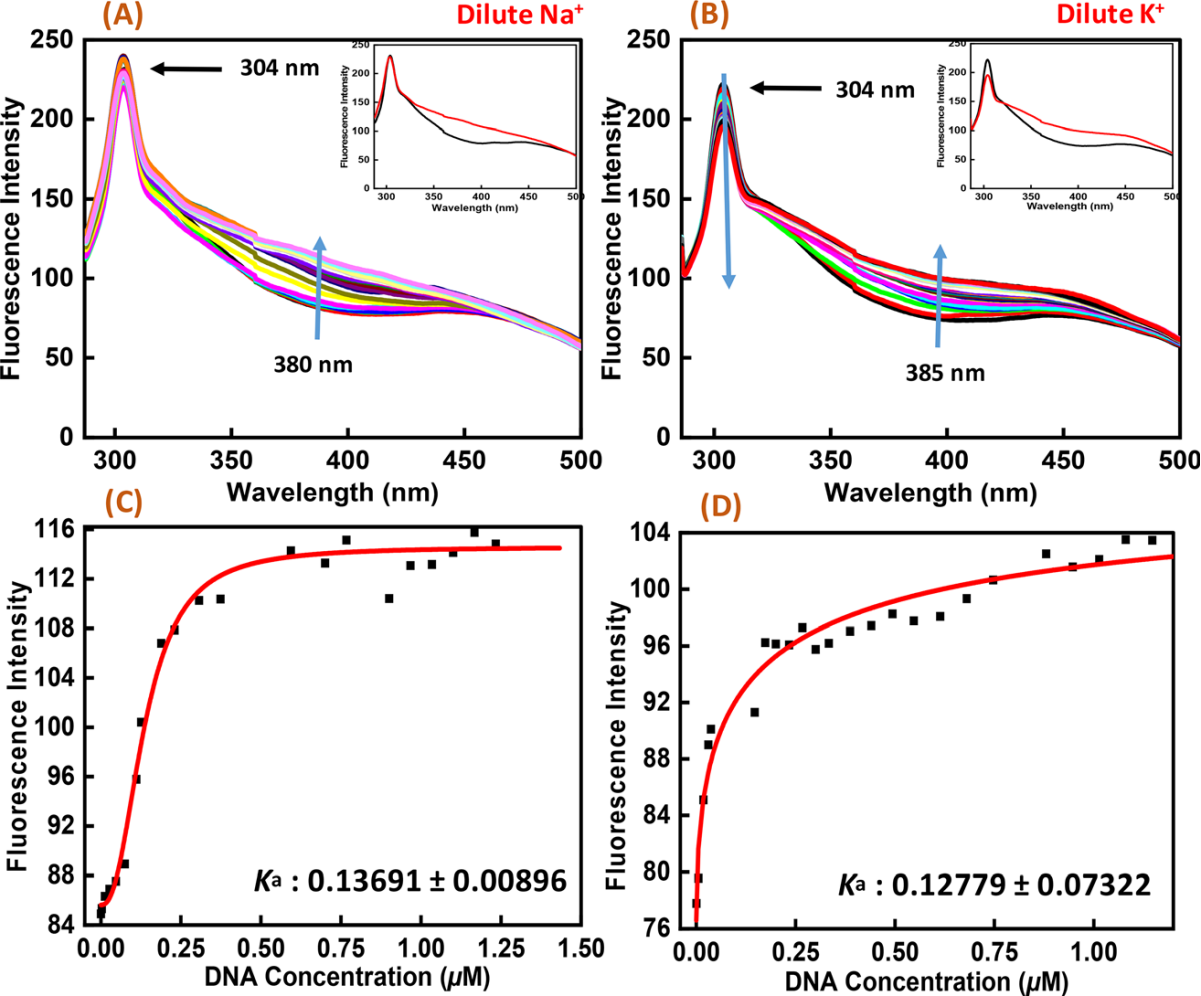
Fluorescence emission spectra of Nu peptide with 4 μM human c-Myc-G4 in 30 mM sodium cacodylate buffer (pH 7.4) containing 0.5 mM EDTA, 100 mM NaCl (A) and 100 mM KCl (B) and their binding constant values (*K*_a_) (C and D) without any additive (black line) and titrated with an increasing concentration of Nu peptide.

Fluorescence spectroscopy was employed to study the binding affinities of Nu peptide with c-Myc promoter G-quadruplex. The fluorescence emission of the Nu peptide was examined in its unbound form at the emission maximum. Upon excitation at 275 nm, the peptide produced an emission band with maxima centered at 304 nm (Fig. 6) because of the presence of proline. The c-Myc promoter G4 prepared in Na^+^ and K^+^ was added to the peptide until very small changes in fluorescence spectra were observed. In addition to DNA, the observed changes in the fluorescence intensity depicted the binding of Nu peptide to the c-Myc promoter G4 and generated a G4-peptide complex. Interestingly, an increase in the fluorescence intensity at was observed at 380 nm in Na^+^ and 385 nm in K^+^ upon increasing the G4 DNA concentration. The value of the binding constant (*K*_a_) was determined by plotting the fluorescence intensity and DNA concentration as 0.1369 ± 0.008 μM and 0.1277 ± 0.073 μM in Na^+^ and K^+^, respectively. The *K*_a_ values confirmed the strong association of Nu peptide with c-Myc G4.

### 3.7 *In vitro* cytotoxic assay of the peptide

**Fig. 7:**
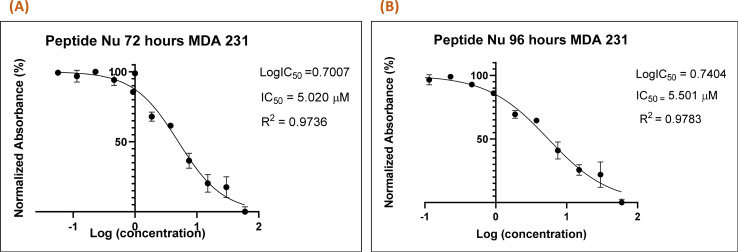
Nu peptide demonstrated the anti-proliferative effects on MDA-MB-231 human breast adenocarcinoma cells. The MTT assay was carried out at two time points (72 h (A) and 96 h (B)), and the difference in the absorbance was due to the different rates of formazan formation for cells incubated with Nu peptide at concentrations ranging from 40 μM to 39 nM. The data were analyzed by comparing the changes in the absorbance from cells with media only (control).

Recently in our previous publication, we reported the anti-proliferative activity of the designed peptide QW10 after binding to the c-Myc promoter G4.^20^ Herein, to evaluate the potential of Nu peptide to stabilize the c-Myc promoter G4 and to check its capacity to inhibit the growth of human breast adenocarcinoma cells MDA-MB-231 cells were examined. For this, we used various time points and a range of concentrations of the peptide were screened to determine the effective time points and peptide concentration(s) with maximum anti-proliferative efficacy of the peptide demonstrating less than 50% cell viability (IC_50_). This technique allowed assessment of the cell viability based on the reduction of the water-soluble, yellow tetrazole salt (MTT) into insoluble dark blue formazan. The amount of reduced MTT is directly proportional to the number of living cells, and the MDA-MB-231 cell viability was significantly decreased in the presence of nucleolin peptide, with IC_50_ values of 5.020 μM and 5.501 μM after 72 and 96 hours, respectively (Fig. 7).

## 4 Conclusion

To conclude, our study offers a novel strategy for targeting G4 structures using naturally occurring peptide segments derived from G4-stabilizing protein. The rationale for choosing c-Myc as the target is its crucial role in promoting cell growth and proliferation. It is overexpressed in a wide range of human cancers, including breast and colon cancers. Research indicates that nucleolin, a multifunctional phosphoprotein, binds with high affinity and selectivity to the c-Myc G-quadruplex compared to other quadruplex structures. Nucleolin acts as a regulator of cell proliferation. Due to the complexities and limitations of using the entire G4-protein for interaction studies—such as due to its complex structure and the difficulty in targeting G4—it is preferable to focus on small molecules to target G4s. Therefore, we identified the specific domain of nucleolin that binds to the G4 and used that 8-mer sequence for interaction studies. The interaction between the Nu peptide and human c-Myc G4 under physiological ionic conditions was thoroughly investigated. Our findings revealed the formation of a parallel G4 structure with significant changes observed in UV thermal and CD melting studies. The strong association between the Nu peptide and c-Myc G4 was confirmed through the calculation of the binding constant, whereby the Nu peptide exhibited preferential cytotoxicity against MDA-MB-231 cells. These results highlight the potential of using peptide-based approaches to modulate G4 structures for therapeutic purposes. Based on our findings, we conclude that our selected peptide has the potential to bind to the c-Myc G4 structure. We propose that this binding occurs through stacking interactions. Although the proline in the peptide lacks an aromatic ring, its pyrrolidine ring structure may facilitate stacking interactions with the bases in the loop region of the c-Myc G4. Additionally, we propose that the binding mode of the Nu peptide with the G4 structure involves interactions with the phosphate groups. As the peptide contains three lysine residues, which is a positively charged amino acid, it may bind to the G4 structures *via* interactions with phosphate groups. Furthermore, the pyrrolidine ring of proline might intercalate within the planes of the G4 structures, enhancing the binding affinity and stability.

## Data availability

The datasets supporting this article have been provided below. All the data presented in this article is present in the form of figures and tables in the manuscript itself.

## Conflicts of interest

The authors declare that there is no conflict of interest regarding the publication of this paper.
